# Prognostic value of albumin to fibrinogen ratio for mortality in patients with hypertrophic cardiomyopathy

**DOI:** 10.1186/s12872-023-03562-8

**Published:** 2023-11-16

**Authors:** Liying Li, Chao Ban, Haiyan Ruan, Muxin Zhang, Ziqiong Wang, Min Ma, Yi Zheng, Sen He

**Affiliations:** 1 Department of Cardiology, West China Hospital of Sichuan University, No.37 Guoxue Alley, Chengdu, China; 2 Department of Equipment, Sichuan University West China Hospital, Chengdu, China; 3 Department of Cardiology, Traditional Chinese Medicine Hospital of Shuangliu District, Chengdu, China; 4 Department of Cardiology, First People’s Hospital, Longquanyi District, Chengdu, China

**Keywords:** Albumin, Fibrinogen, Hypertrophic cardiomyopathy, Prognosis, Inflammation

## Abstract

**Background:**

Albumin to fibrinogen ratio (AFR), a new inflammatory marker, has emerged as a useful indicator to predict adverse outcomes for several diseases. However, whether AFR could be a new useful indicator to predict mortality in HCM patients remains to be evaluated. The study explored the predictive value of AFR for HCM-related death in adult HCM patients.

**Methods:**

A total of 404 HCM patients were eventually enrolled in the study according to the inclusion criteria. Patients were divided into two groups based on the median of baseline AFR. The association between AFR and HCM-related death was analyzed.

**Results:**

During a median follow-up of 4.75 years, HCM-related death was observed in 45 patients (11.1%). The incidence of HCM-related death was significantly higher in the low AFR group (log-rank *p* < 0.001). With the high AFR group as reference, the unadjusted hazard ratio (HR) for HCM-related death was 2.97 (95% confidence interval [CI]: 1.53–5.75, *p* = 0.001) in the low AFR group, and after adjusting for potentially confounding variables, the adjusted HR for low AFR group was 3.15 (95% CI: 1.56–6.37, *p* = 0.001). No significant interactions between AFR and other variables were observed in subgroup analysis. Sensitivity analyses in patients with normal albumin and fibrinogen showed similar results.

**Conclusion:**

AFR is an independent prognostic factor for HCM-related death, adult HCM patients with a lower AFR have a higher risk of HCM-related death.

**Supplementary Information:**

The online version contains supplementary material available at 10.1186/s12872-023-03562-8.

## Introduction

Hypertrophic cardiomyopathy (HCM) is a common inherited heart diseases in the global, mainly caused by pathogenic mutations in genes encoding proteins associated with myocardium and characterized by left ventricular hypertrophy that cannot been explained by physiological factors, heart disease, systemic disease, metabolic disease, or abnormal loading conditions [1, 2]. The prevalence of HCM in the population is approximately 1:500, and people at any age can be diagnosed with this disease [3]. The clinical symptoms of HCM patients various, some patients without any symptoms, while some patients have severe symptoms, such as dyspnea, syncope/pre-syncope, chest pain, and palpitation. Sudden cardiac death (SCD), heart failure, or stroke are the most common adverse outcomes in HCM patients [4]. Although significant advances have been made in the etiology, diagnosis, treatment, and management of HCM over the past few decades, and the overall prognosis of HCM patients have been improved greatly [5], HCM and its related complications remains to bring large burden for patients and healthcare [6]. Therefore, we need to explore more indicators to predict prognosis for HCM patients and to make better risk stratify for those patients who were at high risk of adverse outcomes. Accumulating evidence has found the existence of local or systemic inflammation in HCM in recent years [7–9]. The persistence of inflammatory responses and oxidative stress may result in myocardial fibrosis, which will contribute to myocardial hypertrophy and diastolic dysfunction for HCM patients [10], and then result in adverse outcomes for these patients. Recently, some researches have showed a new inflammatory marker, albumin to fibrinogen ratio (AFR), was associated with adverse outcomes in many diseases, including peritonitis-induced sepsis [11], myocardial infarction [12], ischemic stroke [13], gastric cancer [14], and knee synovitis [15]. However, whether this indicator could be a useful prognostic factor for HCM patients remains unclear. The purpose of the study was to explored the prognostic value of AFR for mortality in Chinese adult HCM patients.

## Methods

### Study patients

**Fig. 1:**
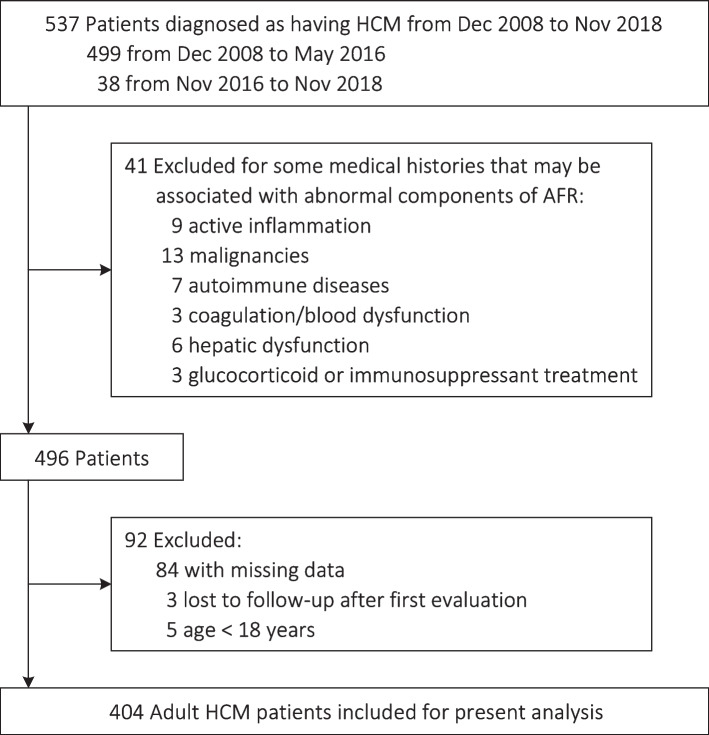
Study flow diagram

This retrospective single-center cohort study was performed at West China Hospital of Sichuan University, a tertiary center hospital located in Chengdu, China. We included all hospitalized patients in our hospital from December 2008 to November 2018 with a discharge diagnosis containing hypertrophic cardiomyopathy (*n* = 546). The diagnosis of HCM is that the presence of one or more left ventricular segments with end-diastolic maximum wall thickness ≥ 15 mm which measured by any cardiac imaging, and the left ventricular outflow tract obstruction (LVOTO) was considered as a gradient ≥ 30 mmHg at rest [16]. When collect data, a researcher first extracted the data from medical records of these patients carefully and then an experienced cardiologist rechecked the data. After rechecking by experienced cardiologist according to the criteria of the European Society of Cardiology [17], 9 patients were excluded: restrictive cardiomyopathy (*n* = 2), cardiac amyloidosis (*n* = 5), myocarditis (*n* = 1) and dilated cardiomyopathy (*n* = 1). Based on the inclusion and exclusion criteria, a total of 404 adult patients were enrolled for the present analysis among the remaining 537 patients (Fig. 1).

The study has been registered on the website of Chinese Clinical Trial Registry (https://www.chictr.org.cn/enIndex.aspx; registration number: ChiCTR2000029352). It was conducted according to the principles of the Declaration of Helsinki, and was approved by the Biomedical Research Ethics Committee, West China Hospital of Sichuan University (approval number: 2019–1147). Since this was a retrospective study, informed consent was waived. Other detailed information has been reported in the recently published article [18, 19].

### Clinical evaluation

We collected these patients' current history, past medical history, the general condition on admission, the results of peripheral blood test, 12-lead electrocardiogram, doppler echocardiography, and the treatment during hospitalization and discharge medication. Cardiac magnetic resonance imaging and other imaging results were also collected as much as possible. All patients had their peripheral venous blood collected by nurses using a tube with EDTA anticoagulant at the time of hospital admission, and the blood samples were sent to the laboratory within 30–60 min. The level of serum albumin was measured by a fully automatic biochemical immunoassay (Roche Cobas 8000), and the fibrinogen measurement was performed by a fully automatic blood clotting analyzer (Sysmex CS-5100). The normal ranges of serum albumin and fibrinogen in our clinical medical laboratory center, which is accredited by the American CAP Medical Laboratory, are 40–55 g/L and 2.0–4.0 g/L, respectively. The AFR was calculated using the following formula: AFR = serum albumin (g/L) / fibrinogen (g/L).

### Study outcomes and follow-up

The study endpoint was HCM-related death, including heart failure (HF)-related death [20], stroke-related death [21, 22], SCD [23], and perioperative death due to ventricular septal myectomy. Follow-up was conducted through medical records or contact with the patients themselves or their relatives by telephone. All patients were followed from the first evaluation up to the endpoint or the most recent evaluation.

### Statistical analysis

Patients were divided into two groups according to the median of baseline AFR, which was evaluated by the receiver operating characteristic (ROC) analysis. ROC analysis revealed that the area under curve of AFR (cut-off = 15.94) to predict HCM-related death was 0.650, and the sensitivity and specificity were 73.3% and 52.9%, respectively (Figure S1). Kruskal–Wallis test, Shapiro–Wilk test, Chi-square test or Fisher exact test were used for data analysis appropriately. For each group, continuous variables were presented as mean ± standard deviation (SD) or median with interquartile range (IQR) appropriately, and categorical variables as number with percentages. Survival curves were constructed using Kaplan–Meier, and the cumulative incidence of HCM-related death was compared using the log-rank test. Cox proportional hazard regression model was applied to perform prognostic analysis. We constructed two multivariate models and variables for inclusion were carefully chosen to ensure parsimony of multivariate models. Model 1, the basic model, adjusted for age and gender. For model 2, we used a backward stepwise modeling approach to select variables, including variables that showed a significant relationship with HCM-related death in univariate analysis (*p* < 0.05) and some clinically relevant variables. Additionally, stratified analysis was performed to assess whether the association between AFR and HCM-related death was consistent in different subgroups. Furthermore, in case of some unknown factors that might affect AFR was not ruled out, we also conducted sensitivity analysis in patients with normal albumin and fibrinogen to assess the relationship between AFR and HCM-related death.

All analyses were performed with R version 4.1.0 (R Project for Statistical Computing). A two-sided *p* value < 0.05 was considered statistically significant.

## Results

### Baseline characteristics

The present study comprised 404 patients (male: 54.64%) with a median age of 57.00 (IQR: 46.00–67.00) years. Patients were divided into two groups: low AFR group (AFR < 15.94) and high AFR group (AFR ≥ 15.94). Baseline characteristics of the two groups are summarized in Table 1 with detailed. Patients in the low AFR group were older and the proportion of women is higher. Patients with hypertension and New York Heart Association III/IV were more common in low AFR group. Compared with patients in the high AFR group, hemoglobin (Hgb), albumin and alanine aminotransferase (ALT) were lower in the low AFR group, while platelet count (PLT), white blood cell count (WBCC) and fibrinogen were higher in these patients. There was no significant difference in family history of HCM, family history of SCD, vascular diseases, medical history, therapy, intervention of obstruction, AST and creatinine between the two groups.

**Table 1:** Baseline characteristics

Variable	All (*n* = 404)	Low AFR (*n* = 202)	High AFR (*n* = 202)	*p* value
Age (years)	57.00 (46.00, 67.00)	60.00 (49.00, 68.75)	53.00 (42.25, 65.00)	< 0.001
Gender, male	220 (54.46%)	95 (47.03%)	125 (61.88%)	0.004
Family history of HCM	36 (8.91%)	19 (9.41%)	17 (8.42%)	0.861
Family history of SCD	14 (3.47%)	6 (2.97%)	8 (3.96%)	0.786
NYHA III-IV	143 (35.40%)	84 (41.58%)	59 (29.21%)	0.013
SBP (mmHg)	120.00 (110.00, 136.00)	120.00 (108.00, 140.00)	120.00 (110.00, 132.00)	0.468
DBP (mmHg)	71.00 (65.00, 80.00)	70.00 (64.00, 80.00)	74.00 (65.25, 80.00)	0.305
Smoke	137 (33.91%)	67 (33.17%)	70 (34.65%)	0.834
Symptom				
Dyspnea	237 (58.66%)	123 (60.89%)	114 (56.44%)	0.419
Chest pain	233 (57.67%)	113 (55.94%)	120 (59.41%)	0.546
Syncope/pre-syncope	136 (33.66%)	65 (32.18%)	71 (35.15%)	0.599
Palpitation	159 (39.36%)	79 (39.11%)	80 (39.60%)	1.000
Medical history				
Prior TE	20 (4.95%)	11 (5.45%)	9 (4.46%)	0.819
Vascular diseases	34 (8.42%)	18 (8.91%)	16 (7.92%)	0.858
Hypertension	130 (32.18%)	75 (37.13%)	55 (27.23%)	0.043
Diabetes	29 (7.18%)	17 (8.42%)	12 (5.94%)	0.441
Atrial fibrillation	77 (19.06%)	35 (17.33%)	42 (20.79%)	0.447
Therapy				
Aspirin	77 (19.06%)	39 (19.31%)	38 (18.81%)	1.000
Clopidogrel	26 (6.44%)	13 (6.44%)	13 (6.44%)	1.000
Warfarin	41 (10.15%)	20 (9.90%)	21 (10.40%)	1.000
Beta blockers	305 (75.50%)	147 (72.77%)	158 (78.22%)	0.247
ACEI or ARB	86 (21.29%)	48 (23.76%)	38 (18.81%)	0.274
Intervention of obstruction				0.134
None	356 (88.12%)	184 (91.09%)	172 (85.15%)	
Alcohol septal ablation	42 (10.40%)	15 (7.43%)	27 (13.37%)	
Septal myectomy	6 (1.49%)	3 (1.49%)	3 (1.49%)	
Device				0.650
None	354 (87.62%)	179 (88.61%)	175 (86.63%)	
Pacemaker	17 (4.21%)	9 (4.46%)	8 (3.96%)	
ICD	33 (8.17%)	14 (6.93%)	19 (9.41%)	
Echocardiographic				
LVEDD (mm)	43.00 (40.00, 47.00)	43.00 (39.00, 47.00)	43.00 (40.00, 47.00)	0.729
LAD (mm)	40.00 (36.00, 45.00)	40.00 (36.00, 45.00)	41.00 (36.00, 45.75)	0.220
MWT (mm)	19.00 (17.00, 22.00)	19.00 (16.00, 21.00)	20.00 (17.00, 23.00)	0.003
LVEF (%)	68.00 (63.00, 72.00)	68.00 (63.00, 72.00)	69.00 (64.00, 72.75)	0.389
Resting LVOTG ≥ 30 mm Hg	172 (42.57%)	86 (42.57%)	86 (42.57%)	1.000
Laboratory tests				
Hgb (g/L)	138.50 (126.75, 151.00)	135.00 (123.00, 146.00)	143.00 (131.00, 154.00)	< 0.001
PLT (10^9^/L)	146.00 (110.00, 186.00)	153.00 (113.00, 198.50)	141.00 (108.25, 173.75)	0.024
WBCC (10^9^/L)	6.31 (5.20, 7.71)	6.46 (5.34, 8.31)	6.12 (5.10, 7.27)	0.017
Albumin (g/L)	42.40 (39.88, 45.02)	41.25 (38.45, 43.40)	43.65 (41.70, 46.30)	< 0.001
ALT (IU/L)	23.00 (16.00, 34.00)	21.00 (15.00, 33.00)	24.00 (18.00, 34.75)	0.020
AST (IU/L)	26.00 (21.00, 32.00)	26.00 (20.00, 33.00)	26.00 (21.00, 32.00)	0.814
Glucose (mmol/L)	5.44 (4.88, 6.43)	5.42 (4.88, 6.53)	5.46 (4.89, 6.33)	0.723
Creatinine (umol/L)	80.60 (67.22, 94.03)	81.00 (66.03, 96.00)	80.25 (68.40, 92.40)	0.837
TG (mmol/L)	1.26 (0.96, 1.87)	1.25 (0.93, 1.70)	1.28 (0.97, 1.98)	0.187
TC (mmol/L)	4.28 (3.56, 4.82)	4.28 (3.64, 4.82)	4.26 (3.50, 4.82)	0.652
HDL-C (mmol/L)	1.27 (1.03, 1.54)	1.29 (1.02, 1.56)	1.26 (1.03, 1.51)	0.668
LDL-C (mmol/L)	2.43 ± 0.76	2.42 ± 0.76	2.44 ± 0.76	0.841
Fibrinogen (g/L)	2.65 (2.26, 3.28)	3.28 (2.87, 3.70)	2.26 (2.02, 2.47)	< 0.001

Values are mean ± SD or median (IQR) or n (%)

*Abbreviations*: *HCM* Hypertrophic cardiomyopathy, *AFR* Albumin to fibrinogen ratio, *SCD* Sudden cardiac death, *NYHA* New York heart association, *SBP* Systolic blood pressure, *DBP* Diastolic blood pressure, *TE* Thrombo-embolic event, *ACEI* Angiotensin-converting enzyme inhibitor, *ARB* Angiotensin receptor blocker, *ICD* Implantable cardioverter defibrillator, *LVEDD* Left ventricular end-diastolic dimension, *LAD* Left atrial diameter, *MWT* Maximal left ventricular wall thickness, *LVEF* Left ventricular ejection fraction, *LVOTG* Left ventricular outflow tract gradient, *Hgb* Hemoglobin, *PLT* Platelet count, *WBCC* White blood cell count, *ALT* Alanine aminotransferase, *AST* Aspartate aminotransferase, *TG* Triglyceride, *TC* Cholesterol, *HDL-C* High density lipoprotein cholesterol, *LDL-C* Low density lipoprotein cholesterol

### Study endpoints

During a median follow-up of 4.75 years (IQR: 2.2–6.8 years), HCM-related death occurred in 45 (11.1%) patients, including 22 (5.4%) HF-related death, 8 (2.0%) stroke-related death, 13 (3.2%) SCD and 2 (0.5%) HCM-related postoperative death.

### Association between AFR and mortality

**Fig. 2:**
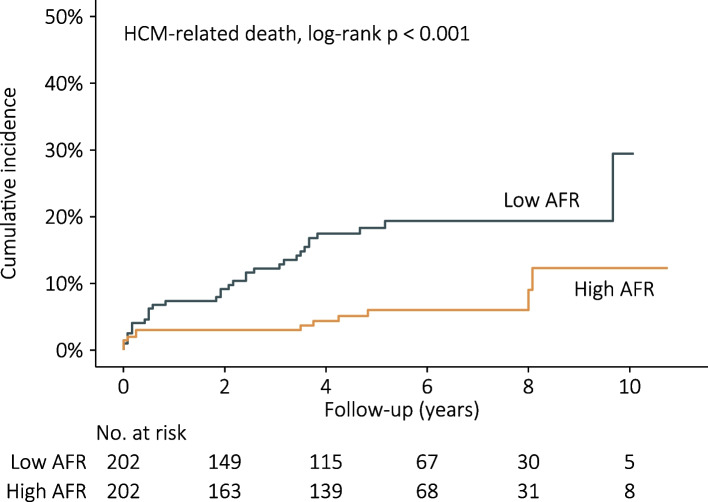
Baseline AFR and HCM-related death by Kaplan–Meier curve analysis. Patients in the low AFR group (AFR < 15.94) had higher incidence of HCM-related death than in the high AFR group (AFR ≥ 15.94) (log-rank *p* < 0.001). HCM: hypertrophic cardiomyopathy; AFR: albumin to fibrinogen ratio

**Fig. 3:**
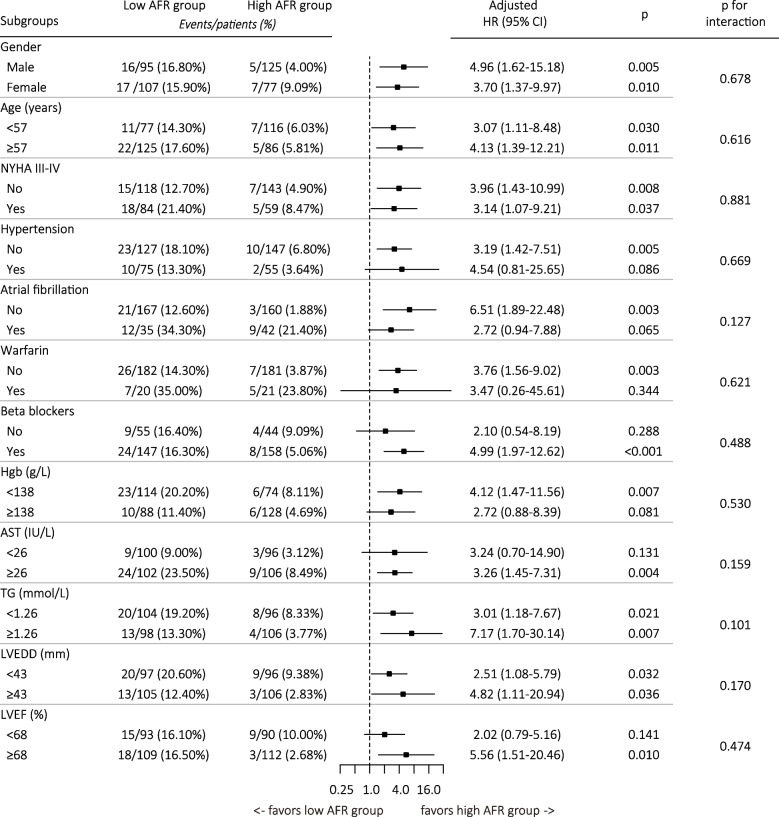
Stratified analyses of HCM-related death. Each stratification adjusted for age, gender, dyspnea, NYHA class, AF, AST, TG, LVEDD, LVEF, resting LVOTG ≥ 30 mm Hg, except the stratification factor itself. The p value for interaction represents the likelihood of interaction between variable and AFR. Abbreviations as in Tables 1 and 2

The cumulative incidence of HCM-related death was significantly higher in the low AFR group (log-rank *p* <0.001) (Fig. 2), as well as HF-related death (log-rank *p* = 0.006) and SCD (log-rank *p* = 0.036) (Figure S2). Univariate Cox regression analysis indicated that AFR and some other variables could predict HCM-related death, with AFR increased per 1 SD, the HR for HCM-related death was decrease by 36% (Table S1). With the high AFR group as reference, the unadjusted HR for HCM-related death was 2.97 (95% CI: 1.53-5.75, *p* = 0.001) in the low AFR group. After adjusting potential confounders, the risk of HCM-related death was approximately three times in the low AFR group than in the high AFR group (fully adjusted HR: 3.15, 95% CI: 1.56-6.37, *p* = 0.001) (Table 2). The results of stratified analysis showed that the mortality rates were consistently higher in the low AFR group in different subgroups and no interaction effect was observed between AFR and other variables for mortality prediction (Fig. 3).

**Table 2:** Associations of AFR and HCM-related death

	High AFR	Low AFR
No. of patients (n)	202	202
Endpoints (n)	12	33
Follow-up (PYs)	995.4	904.5
Mortality rates^a^ (95% CI)	1.2 (0.5–1.9)	3.6 (2.4–4.9)
Unadjusted HRs (95% CI), p	1.00 (ref)	2.97 (1.53–5.75), 0.001
Adjusted HRs (95% CI), p		
model 1	1.00 (ref)	2.80 (1.43–5.48), 0.003
model 2	1.00 (ref)	3.15 (1.56–6.37), 0.001

Model 1 with adjustment for age and gender

Model 2 with adjustment for age, gender, dyspnea, NYHA III-IV, family history of SCD, AF, AST, TG, MWT, and resting LVOTG ≥ 30 mm Hg

*Abbreviations*: *PYs* Person-years, *CI* Confidence interval, *HR* Hazard ratio, other abbreviations as in Table 1

^a^Per 100 PYs

### Sensitivity analysis

Sensitivity analysis was performed in patients with normal albumin and fibrinogen (*n* = 251) and the results were consistent with the main analysis. There were 16 (6.3%) patients occurred in HCM-related death. Kaplan–Meier analysis demonstrated the incidence of HCM-related death was higher in the low AFR group (log-rank *p* = 0.008) (Figure S3). With the high AFR group as reference, the unadjusted HR for HCM-related death was 4.72 (95% CI:1.34–16.62, *p* = 0.016) in the low AFR group. After adjusting potential confounders, the adjusted HR for HCM-related death was 9.21 in the low AFR group (95% CI:1.97–43.10, *p* = 0.005) (Table S2).

### Correlation

AFR was negatively correlated with age (*r* =  − 0.189, *p* < 0.001) and WBCC (*r* =  − 0.102, *p* = 0.041), and positively correlated with Hgb (*r* = 0.140, *p* = 0.005), ALT (*r* = 0.254, *p* < 0.001), AST (*r* = 0.245, *p* < 0.001) and maximal left ventricular wall thickness (*r* = 0.121, *p* = 0.015). In addition, AFR was no correlation with PLT, lipid, left ventricular end-diastolic dimension, left atrial diameter, and left ventricular ejection fraction (Table S3).

## Discussion

The present study showed that AFR was a significant predictor for HCM-related death in adult HCM patients. Stratified analysis in subgroups and sensitivity analysis in patients with normal albumin and fibrinogen demonstrated similar results. To our knowledge, the present study firstly illustrated the prognostic value of AFR in patients with HCM.

In recent years, more and more researches supported the association between inflammation and HCM [7, 8]. Compared with healthy patients, several inflammatory markers in peripheral blood were increased in HCM patients, such as high-sensitivity C-reactive protein (hs-CRP) [9], interleukin-6(IL-6) [24] and tumour necrosis factor-α [25]. Persistent cardiac inflammation will contribute to myocardial fibrosis and remodeling, as well as ventricular diastolic dysfunction [7, 8]. Myocardial fibrosis will cause electromechanical disturbances in the myocardium, and block the supplement of nutrients to the myocardium in some extent, resulting in a vicious cycle between inflammation, fibrosis and myocyte death [8, 26]. Meanwhile, myocardial fibrosis is one of the major determinants of SCD, heart failure and ventricular tachycardia [27–29], which are manifestations of adverse outcomes in patients with HCM. Therefore, inflammatory markers can be used as one of the indicators to assess disease severity and predict adverse outcomes of patients with HCM. In recent years, many studies have explored the relationship between inflammatory markers and the prognosis of HCM patients. Study performed by Burak et al. suggested that monocyte count to high-density lipoprotein cholesterol ratio (MHR) was an independent predictor for prognosis of patients with HCM, patients with higher MHR had higher risk of cardiovascular death and malignant arrhythmic events [30]. Zhu et al. explored the relationship between hs-CRP and the prognosis of HCM patients (*n* = 490), they found that patients with higher plasma hs-CRP have higher risk of adverse outcomes, including cardiovascular death, SCD and all-cause mortality [9]. Our team also found systemic immune-inflammation index (platelet × neutrophil/lymphocyte ratio) was a significant risk factor for all-cause mortality in HCM patients [18].

Albumin is produced primarily by the liver and usually as a negative protein in the acute-phase. Its plasma concentration is mainly influenced by several factors, including the rate of albumin synthesis, catabolism, and loss by renal [31]. Many studies have showed the level of albumin was associated with adverse outcomes of many diseases, including heart failure, atrial fibrillation, ischemic heart disease, myocardial infarction, stroke and venous thromboembolism [31–33]. In our cohort study, the level of AST and creatinine between the two groups was not significantly difference, which indicated there were no significant difference about the liver function and renal function between the two groups patients. Therefore, the main factors affecting serum albumin are inflammation and malnutrition in these patients. In addition, some studies have supported that malnutrition and inflammation play a major role in low level of serum albumin [32, 34] and the influence of inflammation on serum albumin level seems to be stronger [35, 36]. Fibrinogen, a positive acute-phase protein, produced in the liver on the stimulation of IL-6 and some other inflammatory markers, and its levels in plasma will increase with the presence of infection and inflammation [37, 38]. Several studies have demonstrated that plasma fibrinogen is a predictor for cardiovascular disease [39, 40]. AFR, a novel inflammatory biomarker, has been widely proposed as a prognostic marker in various diseases [11–15]. AFR takes serum albumin (a negative acute-phase protein) and fibrinogen (a positive acute-phase protein) into account, it could better reflect the status of inflammation than alone. Gabay et al. [41] found anemia and hypoalbuminemia due to inflammation are common among hospitalized patients. In our study, patients with lower AFR value had lower levels of Hgb and albumin, which also demonstrating the presence of inflammation in these patients indirectly. Despite the lack of diagnostic specificity, AFR is still a useful indicator which reflect the presence of inflammatory process of patients, it could be regarded as a predictor for many diseases which associated with inflammation. Controlling the inflammatory response as much as possible may improve myocardial fibrosis in patients with HCM and reduce the incidence of adverse outcomes.

In this study, the rate of HCM-related death was about 11.1%, and the event rates of SCD, HF-related death, stroke-related death, and HCM-related postoperative death were 3.2%, 5.4%, 2.0%, 0.5%, respectively. In a large longitudinally cohort, which from two American HCM centers (*n* = 1000), Maron et al. found the rate of HCM-related death was 4.0%, and the mortality rates of HF, SCD, embolic stroke, and HCM-related postoperative were 1.7%, 1.7%, 0.2%, 0.4%, respectively [42]. Zhu et al. used database from Fuwai Hospital of China to study the relationship between hs-CRP and the prognosis of HCM patients, they showed the rate of cardiovascular death was 6.1%, including 2.2% SCD, 2.9% HF-related death and 1.0% stroke-related death. The mortality rates between these studies are different, and the likely reasons are the differences in ethnicity and baseline characteristics of the study population. The HCM-related death rate in our study was higher than other studies, which may due to these patients were relatively serious and many patients were referred from local hospitals.

The study has several limitations. Firstly, this was a single center, retrospective study, and those patients were from China, lack of region diversification and race comparison. Secondly, the most widely used inflammatory indicators including the plasma CRP, procalcitonin, erythrocyte sedimentation rate, or IL-6 were not determined, thus the associations between AFR and some well-established inflammation indicators are missing. Thirdly, some important data, including non-sustained ventricular tachycardia and cardiac magnetic resonance (CMR) imaging, were lacking for some patients, which resulting in the association between AFR and HCM Risk-SCD score and the sequences of CMR cannot be explored. Finally, AFR was evaluated only initial evaluation, whether the dynamic changes of AFR could still predict HCM-related death is unclear. However, AFR is a simple and inexpensive routine laboratory test, and it can provide relevant prognostic information for patients with HCM. It could help physician with the risk stratification, facilitate follow-up. Therefore, AFR should serve as a potential screening tool for HCM patients to identify patients at higher risk for adverse outcomes.

## Conclusion

The present study indicated that AFR is an independent prognostic factor for HCM-related death, a lower AFR is associated with increased risk of HCM-related death in adult HCM patients. It is unknown whether prevention and correction of low AFR could improve outcome of patients with HCM, further prospective studies are needed to assess the relationship between AFR and the prognosis of HCM.

### Supplementary Information

**Additional file 1: Figure S1. **ROC analysis revealed that the AUC of AFR (cut-off = 15.94) to predict HCM-related death was 0.650, and the sensitivity was 73.3%, specificity = 52.9%. ROC: Receiver operating characteristic; AUC: Area Under Curve; AFR: albumin to fibrinogen ratio; HCM: hypertrophic cardiomyopathy.

**Additional file 2: Figure S2. **Kaplan–Meier survival curve analysis for HF-related death (A) and SCD (B) by baseline AFR.

**Additional file 3: Figure S3. **Kaplan–Meier survival curve analysis in HCM patients with normal albumin and fibrinogen.

**Additional file 4: Table S1. **Univariate Cox regression analyses for HCM-related death.

**Additional file 5: Table S2. **Associations of AFR and HCM-related death in patients with normal albumin and fibrinogen.

**Additional file 6: Table S3. **Relationships between AFR and other clinical parameters. 

## Data Availability

The datasets used during the current study are available from the corresponding author on reasonable request.

## Abbreviations

HCM: Hypertrophic cardiomyopathy
AFR: Albumin to fibrinogen ratio
SCD: Sudden cardiac death
TE: Thrombo-embolic event
LVEDD: Left ventricular end-diastolic dimension
LAD: Left atrial diameter
MWT: Maximal left ventricular wall thickness
LVEF: Left ventricular ejection fraction
LVOTG: Left ventricular outflow tract gradient
Hgb: Hemoglobin
PLT: Platelet count
WBCC: White blood cell count
HDL-C: High density lipoprotein cholesterol
LDL-C: Low density lipoprotein cholesterol

## References

[1] Ommen SR, Mital S, Burke MA, Day SM, Deswal A, Elliott P, Evanovich LL, Hung J, Joglar JA, Kantor P (2020). 2020 AHA/ACC Guideline for the Diagnosis and Treatment of Patients With Hypertrophic Cardiomyopathy: Executive Summary: A Report of the American College of Cardiology/American Heart Association Joint Committee on Clinical Practice Guidelines. Circulation.

[2] Esposito A, Monda E, Gragnano F, De Simone F, Cesaro A, Natale F, Concilio C, Moscarella E, Caiazza M, Pazzanese V (2020). Prevalence and clinical implications of hyperhomocysteinaemia in patients with hypertrophic cardiomyopathy and MTHFR C6777T polymorphism. Eur J Prev Cardiol.

[3] Maron BJ, Desai MY, Nishimura RA, Spirito P, Rakowski H, Towbin JA, Dearani JA, Rowin EJ, Maron MS, Sherrid MV (2022). Management of Hypertrophic Cardiomyopathy JACC State-of-the-Art Review. J Am Coll Cardiol.

[4] Maron BJ, Maron MS (2013). Hypertrophic cardiomyopathy. Lancet.

[5] Maron BJ, Maron MS, Rowin EJ (2017). Perspectives on the Overall Risks of Living With Hypertrophic Cardiomyopathy. Circulation.

[6] Ho CY, Day SM, Ashley EA, Michels M, Pereira AC, Jacoby D, Cirino AL, Fox JC, Lakdawala NK, Ware JS (2018). Genotype and Lifetime Burden of Disease in Hypertrophic Cardiomyopathy. Circulation.

[7] Becker RC, Owens AP, Sadayappan S (2020). Tissue-level inflammation and ventricular remodeling in hypertrophic cardiomyopathy. J Thromb Thrombolysis.

[8] Monda E, Palmiero G, Rubino M, Verrillo F, Amodio F, Di Fraia F, Pacileo R, Fimiani F, Esposito A, Cirillo A (2020). Molecular Basis of Inflammation in the Pathogenesis of Cardiomyopathies. Int J Mol Sci.

[9] Zhu L, Zou Y, Wang Y, Luo X, Sun K, Wang H, Jia L, Liu Y, Zou J, Yuan Z (2017). Prognostic Significance of Plasma High-Sensitivity C-Reactive Protein in Patients With Hypertrophic Cardiomyopathy. J Am Heart Assoc.

[10] Fang L, Ellims AH, Beale AL, Taylor AJ, Murphy A, Dart AM (2017). Systemic inflammation is associated with myocardial fibrosis, diastolic dysfunction, and cardiac hypertrophy in patients with hypertrophic cardiomyopathy. Am J Transl Res.

[11] Tai H, Zhu Z, Mei H, Sun W, Zhang W (2020). Albumin-to-Fibrinogen Ratio Independently Predicts 28-Day Mortality in Patients with Peritonitis-Induced Sepsis. Mediators Inflamm.

[12] Xiao L, Jia Y, Wang X, Huang H (2019). The impact of preoperative fibrinogen-albumin ratio on mortality in patients with acute ST-segment elevation myocardial infarction undergoing primary percutaneous coronary intervention. Clin Chim Acta.

[13] Ruan Y, Yuan C, Liu Y, Zeng Y, Cheng H, Cheng Q, Chen Y, Huang G, He W, He J (2021). High fibrinogen-to-albumin ratio is associated with hemorrhagic transformation in acute ischemic stroke patients. Brain Behav.

[14] You X, Zhou Q, Song J, Gan L, Chen J, Shen H (2019). Preoperative albumin-to-fibrinogen ratio predicts severe postoperative complications in elderly gastric cancer subjects after radical laparoscopic gastrectomy. BMC Cancer.

[15] Gao K, Zhang Y, Sun S, Lin B, Liu W, Lai W, Wu Y, Lin Z, Jiang Y, Cao Y (2021). Novel inflammatory markers in the blood of patients with knee synovitis. J Int Med Res.

[16] Lang RM, Badano LP, Mor-Avi V, Afilalo J, Armstrong A, Ernande L, Flachskampf FA, Foster E, Goldstein SA, Kuznetsova T (2015). Recommendations for Cardiac Chamber Quantification by Echocardiography in Adults: An Update from the American Society of Echocardiography and the European Association of Cardiovascular Imaging. J Am Soc Echocar.

[17] Elliott PM, Anastasakis A, Borger MA, Borggrefe M, Cecchi F, Charron P, Hagege AA, Lafont A, Limongelli G, Mahrholdt H (2014). 2014 ESC Guidelines on diagnosis and management of hypertrophic cardiomyopathy The Task Force for the Diagnosis and Management of Hypertrophic Cardiomyopathy of the European Society of Cardiology (ESC). Eur Heart J.

[18] Wang Z, Ruan H, Li L, Wei X, Zhu Y, Wei J, Chen X, He S. Assessing the relationship between systemic immune-inflammation index and mortality in patients with hypertrophic cardiomyopathy. Ups J Med Sci. 2021;126:e8124. 10.48101/ujms.v126.8124. 10.48101/ujms.v126.8124 PMC8693584 34984097

[19] Liao H, Wang Z, Zhao L, Chen X, He S (2020). Myocardial contraction fraction predicts mortality for patients with hypertrophic cardiomyopathy. Sci Rep.

[20] Coats CJ, Gallagher MJ, Foley M, Omahony C, Critoph C, Gimeno J, Dawnay A, McKenna WJ, Elliott PM (2013). Relation between serum N-terminal pro-brain natriuretic peptide and prognosis in patients with hypertrophic cardiomyopathy. Eur Heart J.

[21] Haruki S, Minami Y, Hagiwara N (2016). Stroke and Embolic Events in Hypertrophic Cardiomyopathy Risk Stratification in Patients Without Atrial Fibrillation. Stroke.

[22] Maron BJ, Olivotto I, Bellone P, Conte MR, Cecchi F, Flygenring BP, Casey SA, Gohman TE, Bongioanni S, Spirito P (2002). Clinical profile of stroke in 900 patients with hypertrophic cardiomyopathy. J Am Coll Cardiol.

[23] Elliott PM, Poloniecki J, Dickie S, Sharma S, Monserrat L, Varnava A, Mahon NG, McKenna WJ (2000). Sudden death in hypertrophic cardiomyopathy: Identification of high risk patients. J Am Coll Cardiol.

[24] Hogye M, Mandi Y, Csanady M, Sepp R, Buzas K (2004). Comparison of circulating levels of interleukin-6 and tumor necrosis factor-alpha in hypertrophic cardiomyopathy and in idiopathic dilated cardiomyopathy. Am J Cardiolo.

[25] Zen K, Irie H, Doue T, Takamiya M, Yamano T, Sawada T, Azuma A, Matsubara H (2005). Analysis of circulating apoptosis mediators and proinflammatory cytokines in patients with idiopathic hypertrophic cardiomyopathy - Comparison between nonobstructive and dilated-phase hypertrophic cardiomyopathy. Int Heart J.

[26] Piek A, de Boer RA, Sillje HHW (2016). The fibrosis-cell death axis in heart failure. Heart Fail Rev.

[27] Ho CY, Lopez B, Coelho-Filho OR, Lakdawala NK, Cirino AL, Jarolim P, Kwong R, Gonzalez A, Colan SD, Seidman JG (2010). Myocardial Fibrosis as an Early Manifestation of Hypertrophic Cardiomyopathy. N Engl J Med.

[28] Varnava AM, Elliott PM, Sharma S, McKenna WJ, Davies MJ (2000). Hypertrophic cardiomyopathy: the interrelation of disarray, fibrosis, and small vessel disease. Heart.

[29] Adabag AS, Maron BJ, Appelbaum E, Harrigan CJ, Buros JL, Gibson CM, Lesser JR, Hanna CA, Udelson JE, Manning WJ (2008). Occurrence and frequency of arrhythmias in hypertrophic cardiomyopathy on relation to delayed enhancement on cardiovascular magnetic resonance. J Am Coll Cardiol.

[30] Ekizler FA, Cay S, Acar B, Tak BT, Kafes H, Ozeke O, Cetin EHO, Ozcan F, Topaloglu S, Tufekcioglu O (2019). Monocyte to high-density lipoprotein cholesterol ratio predicts adverse cardiac events in patients with hypertrophic cardiomyopathy. Biomark Med.

[31] Arques S (2018). Human serum albumin in cardiovascular diseases. Eur J Intern Med.

[32] Arques S, Ambrosi P (2011). Human Serum Albumin in the Clinical Syndrome of Heart Failure. J Card Fail.

[33] Djousse L, Rothman KJ, Cupples LA, Levy D, Ellison RC (2002). Serum albumin and risk of myocardial infarction and all-cause mortality in the Framingham Offspring Study. Circulation.

[34] Wada H, Dohi T, Miyauchi K, Shitara J, Endo H, Doi S, Naito R, Konishi H, Tsuboi S, Ogita M (2017). Impact of serum albumin levels on long-term outcomes in patients undergoing percutaneous coronary intervention. Heart Vessels.

[35] Eckart A, Struja T, Kutz A, Baumgartner A, Baumgartner T, Zurfluh S, Neeser O, Huber A, Stanga Z, Mueller B (2020). Relationship of Nutritional Status, Inflammation, and Serum Albumin Levels During Acute Illness: A Prospective Study. Am J Med.

[36] Kaysen GA, Dubin JA, Muller HG, Rosales L, Levin NW, Mitch WE, Grp HS (2004). Inflammation and reduced albumin synthesis associated with stable decline in serum albumin in hemodialysis patients. Kidney Int.

[37] Tennent GA, Brennan SO, Stangou AJ, O'Grady J, Hawkins PN, Pepys MB (2007). Human plasma fibrinogen is synthesized in the liver. Blood.

[38] Yamaguchi T, Yamamoto Y, Yokota S, Nakagawa M, Ito M, Ogura T (1998). Involvement of interleukin-6 in the elevation of plasma fibrinogen levels in lung cancer patients. Jpn J Clin Oncol.

[39] Deveci B, Gazi E (2021). Relation Between Globulin, Fibrinogen, and Albumin With the Presence and Severity of Coronary Artery Disease. Angiology.

[40] Kannel WB, Wolf PA, Castelli WP, Dagostino RB (1987). Fibrinogen and risk of cardiovascular disease. The Framingham Study JAMA.

[41] Gabay C, Kushner I (1999). Mechanisms of disease: Acute-phase proteins and other systemic responses to inflammation. N Engl J Med.

[42] Maron BJ, Rowin EJ, Casey SA, Link MS, Lesser JR, Chan RHM, Garberich RF, Udelson JE, Maron MS (2015). Hypertrophic Cardiomyopathy in Adulthood Associated With Low Cardiovascular Mortality With Contemporary Management Strategies. J Am Coll Cardiol.

