# Supplementary material for: Prognostic value of albumin to fibrinogen ratio for mortality in patients with hypertrophic cardiomyopathy
Source: BMC Cardiovasc Disord. 2023 Nov 16;23:559. doi: 10.1186/s12872-023-03562-8 (PMC10652625; doi:10.1186/s12872-023-03562-8)
Supplement: Supplementary file 1 — Additional file 1: Figure S1. ROC analysis revealed that the AUC of AFR (cut-off = 15.94) to predict HCM-related death was 0.650, and the sensitivity was 73.3%, specificity = 52.9%. ROC: Receiver operating characteristic; AUC: Area Under Curve; AFR: albumin to fibrinogen ratio; HCM: hypertrophic cardiomyopathy. [file 12872_2023_3562_MOESM1_ESM.docx]

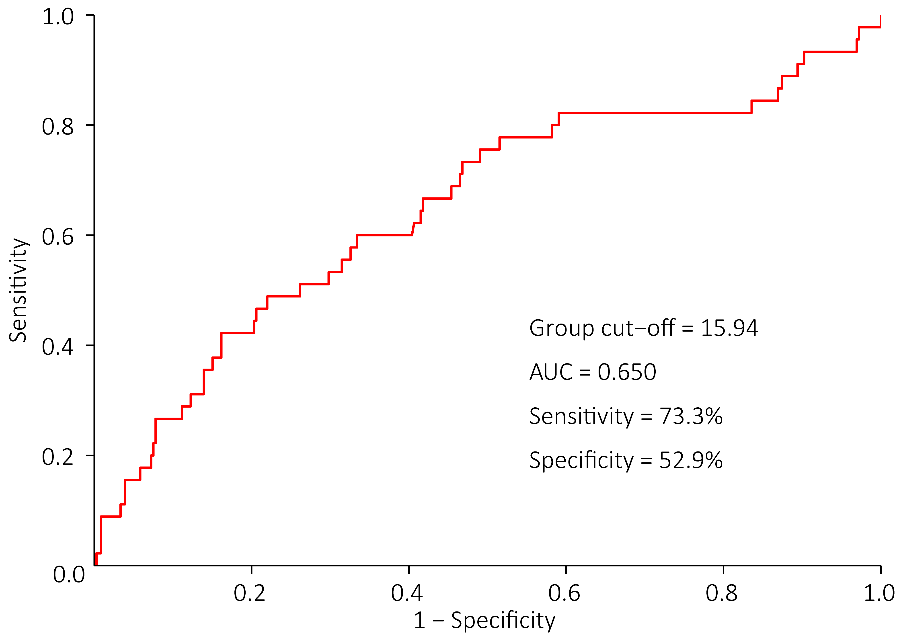


**Figure S1.** ROC analysis revealed that the AUC of AFR (cut-off = 15.94) to predict HCM-related death was 0.650, and the sensitivity was 73.3%, specificity = 52.9%. ROC: Receiver operating characteristic; AUC: Area Under Curve; AFR: albumin to fibrinogen ratio; HCM: hypertrophic cardiomyopathy.

.
