# Supplementary material for: Prognostic value of albumin to fibrinogen ratio for mortality in patients with hypertrophic cardiomyopathy
Source: BMC Cardiovasc Disord. 2023 Nov 16;23:559. doi: 10.1186/s12872-023-03562-8 (PMC10652625; doi:10.1186/s12872-023-03562-8)
Supplement: Supplementary file 2 — Additional file 2: Figure S2. Kaplan–Meier survival curve analysis for HF-related death (A) and SCD (B) by baseline AFR. [file 12872_2023_3562_MOESM2_ESM.docx]

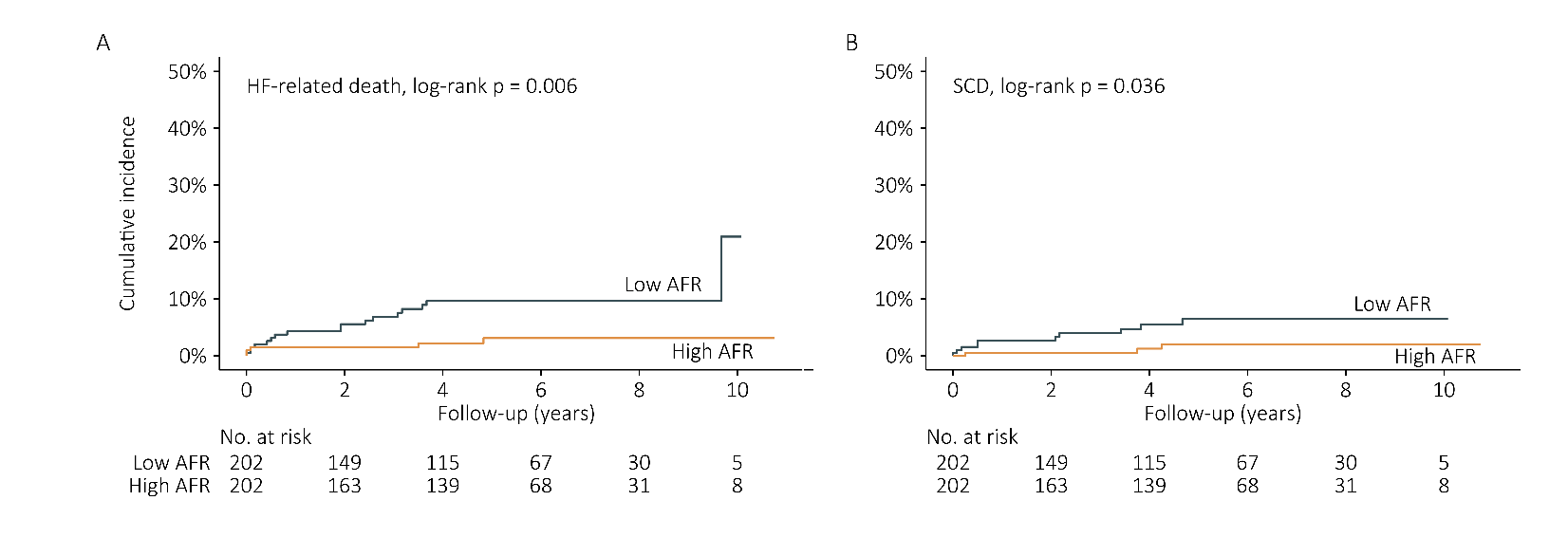


**Figure S2.** Kaplan–Meier survival curve analysis for HF-related death (A) and SCD (B) by baseline AFR.

AFR: albumin to fibrinogen ratio; HF: heart failure; SCD: sudden cardiac death.
