# Supplementary material for: Prognostic value of albumin to fibrinogen ratio for mortality in patients with hypertrophic cardiomyopathy
Source: BMC Cardiovasc Disord. 2023 Nov 16;23:559. doi: 10.1186/s12872-023-03562-8 (PMC10652625; doi:10.1186/s12872-023-03562-8)
Supplement: Supplementary file 3 — Additional file 3: Figure S3. Kaplan–Meier survival curve analysis in HCM patients with normal albumin and fibrinogen. [file 12872_2023_3562_MOESM3_ESM.docx]

**
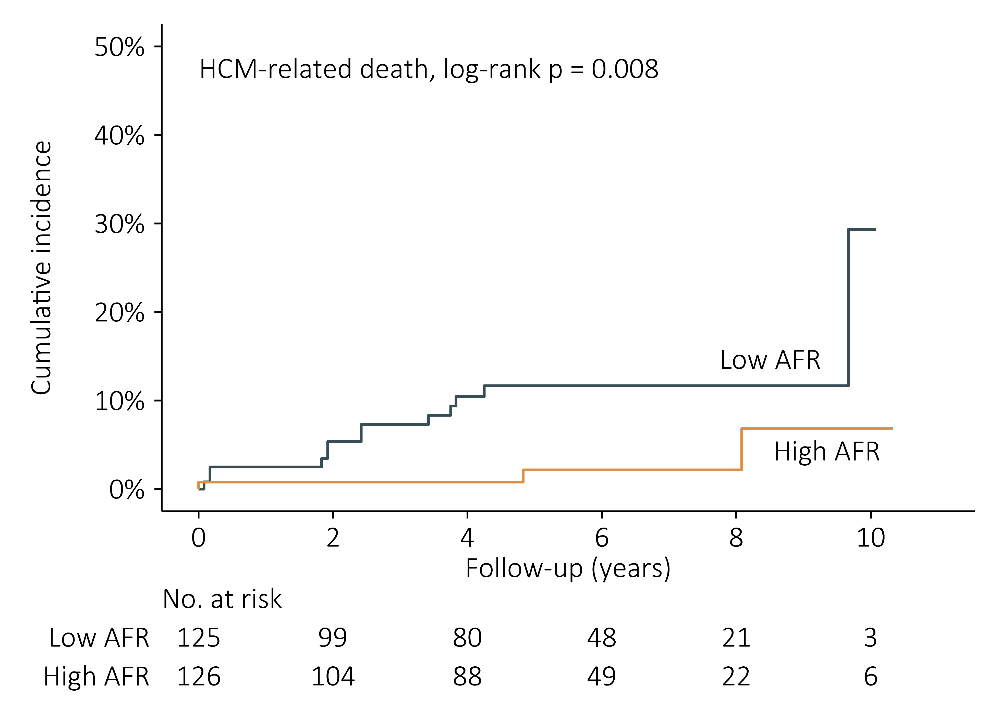
**

**Figure S3.** Kaplan–Meier survival curve analysis in HCM patients with normal albumin and fibrinogen.

Patients in the low AFR group had higher incidence of HCM-related death than those in the high AFR group (log-rank p = 0.008). HCM: hypertrophic cardiomyopathy; AFR: albumin to fibrinogen ratio.
