# Supplementary material for: Prognostic value of albumin to fibrinogen ratio for mortality in patients with hypertrophic cardiomyopathy
Source: BMC Cardiovasc Disord. 2023 Nov 16;23:559. doi: 10.1186/s12872-023-03562-8 (PMC10652625; doi:10.1186/s12872-023-03562-8)
Supplement: Supplementary file 4 — Additional file 4: Table S1. Univariate Cox regression analyses for HCM-related death. [file 12872_2023_3562_MOESM4_ESM.docx]

| **Table S1.** Univariate Cox regression analyses for HCM-related death. | | | |
| --- | --- | --- | --- |
| Variables | Change | HRs (95% CI) | p value |
| Gender | female VS. male | 1.32 (0.73-2.37) | 0.354 |
| Age (years) | per 1 SD increase | 1.24 (0.91-1.69) | 0.172 |
| Family history of HCM | yes VS. no | 1.10 (0.43-2.79) | 0.840 |
| Family history of SCD | yes VS. no | 1.18 (0.28-4.86) | 0.822 |
| NYHA III-IV | yes VS. no | 2.30 (1.28-4.14) | 0.005 |
| SBP (mmHg) | per 1 SD increase | 0.60 (0.42-0.85) | 0.004 |
| DBP (mmHg) | per 1 SD increase | 0.87 (0.65-1.18) | 0.375 |
| Smoke | yes VS. no | 1.09 (0.59-2.01) | 0.779 |
| Dyspnea | yes VS. no | 2.99 (1.44-6.20) | 0.003 |
| Chest pain | yes VS. no | 0.72 (0.40-1.30) | 0.279 |
| Syncope/pre-syncope | yes VS. no | 1.10 (0.60-2.02) | 0.751 |
| Palpitation | yes VS. no | 0.92 (0.51-1.67) | 0.777 |
| Prior TE | yes VS. no | 3.19 (1.35-7.55) | 0.008 |
| Vascular diseases | yes VS. no | 1.16 (0.42-3.26) | 0.772 |
| Hypertension | yes VS. no | 0.78 (0.40-1.51) | 0.461 |
| Diabetes | yes VS. no | 0.56 (0.14-2.32) | 0.427 |
| Atrial fibrillation | yes VS. no | 3.85 (2.14-6.92) | <0.001 |
| Aspirin | yes VS. no | 0.91 (0.42-1.95) | 0.801 |
| Warfarin | yes VS. no | 3.52 (1.82-6.83) | <0.001 |
| Beta blockers | yes VS. no | 0.84 (0.44-1.60) | 0.600 |
| ACEI or ARB | yes VS. no | 0.82 (0.38-1.76) | 0.608 |
| Intervention of obstruction |  |  |  |
| None |  | 1.00(ref) | 1.00(ref) |
| Alcohol septal ablation | yes VS. no | 0.23 (0.03-1.67) | 0.145 |
| Septal myectomy | yes VS. no | 1.73 (0.24-12.6) | 0.589 |
| Device |  |  |  |
| None |  | 1.00(ref) | 1.00(ref) |
| Pacemaker | yes VS. no | 1.07 (0.26-4.44) | 0.925 |
| ICD | yes VS. no | 0.96 (0.34-2.70) | 0.940 |
| LVEDD (mm) | per 1 SD increase | 0.68 (0.49-0.96) | 0.030 |
| LAD (mm) | per 1 SD increase | 1.58 (1.21-2.06) | 0.001 |
| MWT (mm) | per 1 SD increase | 0.91 (0.67-1.23) | 0.533 |
| LVEF (%) | per 1 SD increase | 0.71 (0.56-0.90) | 0.005 |
| Resting LVOTG ≥30 mm Hg | yes VS. no | 1.08 (0.60-1.95) | 0.806 |
| Hgb (g/L) | per 1 SD increase | 0.80 (0.61-1.05) | 0.114 |
| PLT (10^9^/L) | per 1 SD increase | 0.95 (0.70-1.29) | 0.751 |
| WBCC (10^9^/L) | per 1 SD increase | 1.10 (0.84-1.44) | 0.481 |
| Albumin (g/L) | per 1 SD increase | 0.42 (0.33-0.53) | <0.001 |
| Fibrinogen (g/L) | per 1 SD increase | 1.23 (0.96-1.57) | 0.100 |
| AFR | per 1 SD increase | 0.64 (0.45-0.93) | 0.018 |
| ALT (IU/L) | per 1 SD increase | 1.30 (1.06-1.58) | 0.011 |
| AST (IU/L) | per 1 SD increase | 1.32 (1.15-1.51) | <0.001 |
| Glucose (mmol/L) | per 1 SD increase | 1.09 (0.84-1.40) | 0.530 |
| Creatinine (μmol/L) | per 1 SD increase | 1.06 (0.86-1.30) | 0.611 |
| TG (mmol/L) | per 1 SD increase | 0.61 (0.38-0.99) | 0.045 |
| TC (mmol/L) | per 1 SD increase | 0.66 (0.48-0.91) | 0.011 |
| HDL-C (mmol/L) | per 1 SD increase | 0.84 (0.61-1.16) | 0.282 |
| LDL-C (mmol/L) | per 1 SD increase | 0.77 (0.57-1.05) | 0.094 |

Abbreviations as in tables 1 and 2.
