# Supplementary material for: Prognostic value of albumin to fibrinogen ratio for mortality in patients with hypertrophic cardiomyopathy
Source: BMC Cardiovasc Disord. 2023 Nov 16;23:559. doi: 10.1186/s12872-023-03562-8 (PMC10652625; doi:10.1186/s12872-023-03562-8)
Supplement: Supplementary file 5 — Additional file 5: Table S2. Associations of AFR and HCM-related death in patients with normal albumin and fibrinogen. [file 12872_2023_3562_MOESM5_ESM.docx]

| **Table S2.** Associations of AFR with HCM-related death in patients with normal albumin and fibrinogen. | | |
| --- | --- | --- |
|  | High AFR | Low AFR |
| No. of patients (n) | 126 | 125 |
| Endpoints (n) | 3 | 13 |
| Follow-up (PYs) | 652.6 | 613.8 |
| Mortality rates^⁎^ (95% CI) | 0.5 (0.1-1.0) | 2.1 (1.0-3.3) |
| Unadjusted HR (95% CI), p | 1.00 (ref) | 4.72 (1.34-16.62), 0.016 |
| Adjusted HR (95% CI), p |  |  |
| model 1 | 1.00 (ref) | 5.10 (1.44-18.08), 0.012 |
| model 2 | 1.00 (ref) | 9.21 (1.97-43.10), 0.005 |

^⁎^Per 100 PYs.

Model 1 with adjustment for age and gender.

Model 2 with adjustment for age, gender, dyspnea, NYHA III-IV, family history of SCD, AF, AST, TG, MWT, and resting LVOTG.

Abbreviations as in tables 1 and 2.
