# Supplementary material for: Prognostic value of albumin to fibrinogen ratio for mortality in patients with hypertrophic cardiomyopathy
Source: BMC Cardiovasc Disord. 2023 Nov 16;23:559. doi: 10.1186/s12872-023-03562-8 (PMC10652625; doi:10.1186/s12872-023-03562-8)
Supplement: Supplementary file 6 — Additional file 6: Table S3. Relationships between AFR and other clinical parameters. [file 12872_2023_3562_MOESM6_ESM.docx]

| **Table S3.** Relationships between AFR and other clinical parameters. | | |
| --- | --- | --- |
|  | r | p value |
| Age | -0.189 | <0.001 |
| Hgb (g/L) | 0.140 | 0.005 |
| PLT (10^9^/L) | -0.097 | 0.051 |
| WBCC (10^9^/L) | -0.102 | 0.041 |
| ALT (IU/L) | 0.254 | <0.001 |
| AST (IU/L) | 0.245 | <0.001 |
| TG (mmol/L) | -0.011 | 0.824 |
| TC (mmol/L) | -0.033 | 0.511 |
| HDL-C (mmol/L) | -0.028 | 0.575 |
| LDL-C (mmol/L) | -0.010 | 0.843 |
| LVEDD (mm) | 0.005 | 0.919 |
| LAD (mm) | 0.085 | 0.088 |
| MWT (mm) | 0.121 | 0.015 |
| LVEF (%) | -0.006 | 0.898 |

Abbreviations: Hgb: hemoglobin; PLT: Platelet count; WBCC: white blood cell count; ALT: alanine aminotransferase; AST: aspartate aminotransferase; TG: triglyceride; TC: Cholesterol; HDL-C: high density lipoprotein cholesterol; LDL-C: low density lipoprotein cholesterol; LVEDD: left ventricular end-diastolic dimension; LAD: left atrial diameter; MWT: maximal left ventricular wall thickness; LVEF: left ventricular ejection fraction.
